# Scalable Spatial-Spectral Multiplexing of Single-Virus Detection Using Multimode Interference Waveguides

**DOI:** 10.1038/s41598-017-12487-0

**Published:** 2017-09-22

**Authors:** Damla Ozcelik, Aadhar Jain, Alexandra Stambaugh, Matthew A. Stott, Joshua W. Parks, Aaron Hawkins, Holger Schmidt

**Affiliations:** 1 School of Engineering, University of California Santa Cruz, 1156 High Street, Santa Cruz, CA 95064 USA; 2 ECEn Department, 459 Clyde Building, Brigham Young University, Provo, UT 84602 USA

## Abstract

Simultaneous detection of multiple pathogens and samples (multiplexing) is one of the key requirements for diagnostic tests in order to enable fast, accurate and differentiated diagnoses. Here, we introduce a novel, highly scalable, photonic approach to multiplex analysis with single virus sensitivity. A solid-core multimode interference (MMI) waveguide crosses multiple fluidic waveguide channels on an optofluidic chip to create multi-spot excitation patterns that depend on both the wavelength and location of the channel along the length of the MMI waveguide. In this way, joint spectral and spatial multiplexing is implemented that encodes both spatial and spectral information in the time dependent fluorescence signal. We demonstrate this principle by using two excitation wavelengths and three fluidic channels to implement a 6x multiplex assay with single virus sensitivity. High fidelity detection and identification of six different viruses from a standard influenza panel is reported. This multimodal multiplexing strategy scales favorably to large numbers of targets or large numbers of clinical samples. Further, since single particles are detected unbound in flow, the technique can be broadly applied to direct detection of any fluorescent target, including nucleic acids and proteins.

## Introduction

Infectious diseases are responsible for a large fraction of fatalities around the world, accounting for more than 8% of all deaths^1^. Early diagnosis and treatment are essential for preventing the transmission of the infectious agent and development of long-term complications^2^. Therefore, advancement of sensitive and accurate diagnostics systems with low complexity for use in low-resource settings is crucial. However, it is a challenge to accurately diagnose infectious diseases due to low natural concentration levels of the pathogens and their similar early symptoms. Current diagnostic tools, such as real-time polymerase chain reaction (RT-PCR), amplify the small amounts of target nucleic acids of the pathogens to be able to detect them^3–5^. Even though this technique is highly sensitive and specific, it is limited to one analyte type (nucleic acids) and not ideal for rapid point-of-care detection, since it requires complex procedures. On the other hand, eliminating the amplification step requires highly sensitive devices that are capable of reliable sensing of low concentration targets.

In addition to a low limit of detection and large dynamic range, multiplex analysis is another key element of many diagnostic panels as different diseases can present with similar symptoms. Standard tests for influenza or other respiratory infections, for example, simultaneously screen for 4–8 pathogen types^6,7^.

Optofluidic approaches that seek to apply integrated photonic principles to biosensing, bioanalysis, and other applications have produced a number of promising devices^8–10^. In the past couple of years, several optofluidic approaches have shown the capability to optically detect single biological nanoparticles such as viral particles (virions). These include resonance shifts in microspheres upon virus binding^11^, interferometric imaging of viruses bound to a functionalized surface^12^, and fluorescence imaging of virions using a smartphone camera^13^. However, despite their single particle sensitivity, implementing the other requirements of a diagnostic device (low limit of detection, large dynamic range, target specificity, and multiplexed differentiated analysis) is not as straightforward in these approaches.

We have developed a biophotonic analysis platform based on Antiresonant Reflecting Optical Waveguides (ARROWs)^14,15^. Intersecting liquid-core and solid-core ARROWs allow for detection of single biomolecules in flow down to individual nucleic acids at clinically relevant concentrations^16–18^. Moreover, these silicon-based chips can be integrated with advanced sample preparation steps on dedicated microfluidic chips, enabling full sample-to-answer analysis of molecular biomarkers on a chip-based system^17,19,20^. In order to provide multiplexing capabilities, we recently introduced a new paradigm – optofluidic wavelength division multiplexing^21^. By replacing the single-mode solid-core excitation waveguide with an integrated multi-mode interference (MMI) waveguide^22^, well-defined multi-spot excitation patterns were created in a *single* fluidic channel over the entire visible optical bandwidth^21^. With proper design, different patterns can be generated by different excitation wavelengths that are matched to the absorption spectrum of fluorescent labels. This spatio-temporal encoding of spectral information allowed for direct identification of three different influenza strains labeled with different fluorescent tags with extremely high sensitivity and fidelity at clinically relevant concentrations. As an added benefit, the multi-spot excitation approach increases the signal-to-noise ratio and sensitivity of these biosensors, with a SNR enhancement of up to over 50,000 reported in experiments for 8 spot excitation^23^. However, this spectral multiplexing capability is ultimately limited by the finite absorption bandwidth of the dye labels which can lead to channel cross-talk if the absorption spectra overlap. While a further increase of the multiplexing capacity was demonstrated using combinatorial multi-dye labeling^21^, this approach still faces challenges for scaling to large target numbers.

**Figure 1:**
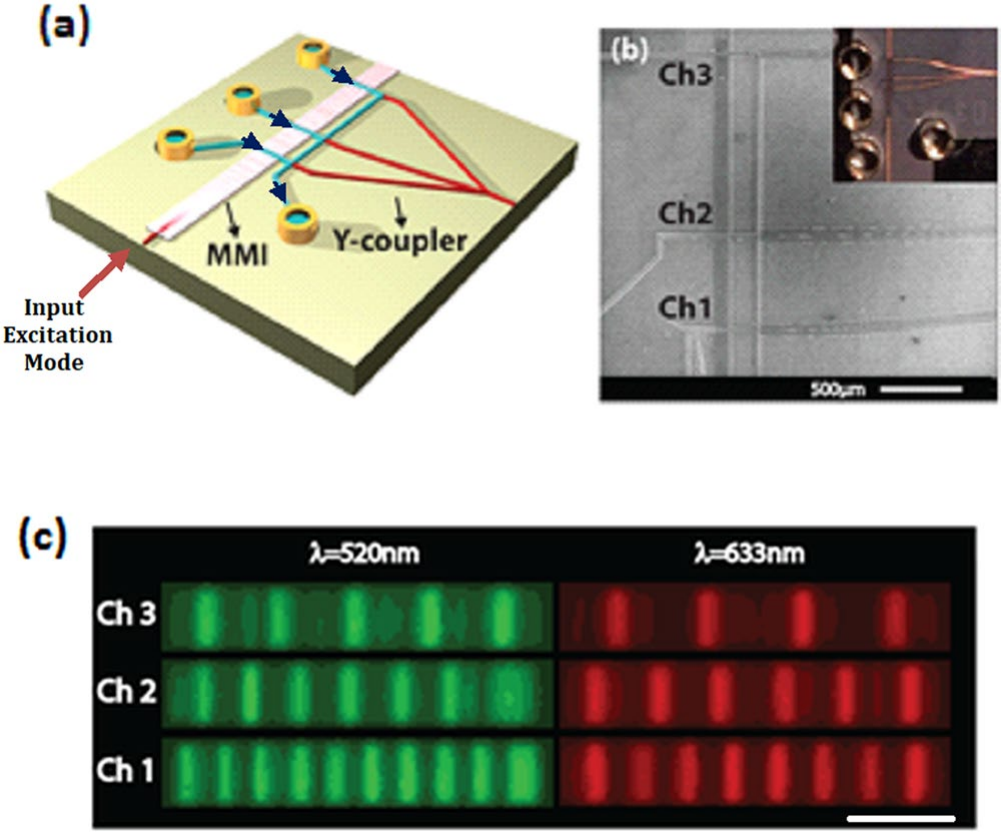
(**a**) Schematic view of the optofluidic chip with MMI waveguide intersecting 3 separate liquid-core waveguides, and a Y-coupler to combine all the signals. The black arrows indicate the direction of flow (**b**) SEM image of the MMI section crossing 3 liquid-core waveguides. Inset: Full optofluidic device with reservoirs attached. (**c**) Images of the excitation patterns in 3 different channels for 2 wavelengths. The channels are filled with fluorescent liquid for imaging. The scale bar is 25 µm.

Here, we introduce a new photonic approach to multiplex detection of single biomolecules. By using a *single* MMI waveguide that crosses *multiple* fluidic channels (see Fig. 1a), we can simultaneously implement both *spectral* and *spatial* multiplexing. We demonstrate the enhanced multiplexing capacity of this device with 6x multiplexing using a combination of two excitation wavelengths and three sample channels. We apply the technique to high-fidelity differentiated detection of single viruses corresponding to a commercial influenza panel. This new type of optofluidic chip provides several options for scaling to large target numbers as well as providing a method for parallel analysis of one or multiple biological samples.

## Device design

A wide solid-core optical waveguide that acts as a multi-mode interference (MMI) waveguide is excited by a narrow single-mode waveguide to create location dependent multi-spot patterns for three orthogonally intersecting liquid-core waveguides (Fig. 1a). This allows for particles flowing through all three channels to be excited using a single MMI section. The SEM image in Fig. 1b shows the central part of the chip where the MMI waveguide crosses the 3 separate liquid-core waveguides. A Y-coupler then combines signals from all 3 channels into a single collection waveguide which is read by an avalanche photo detector (APD). As will be discussed in detail below, since all spectral and spatial information is encoded in the different excitation patterns and it is thus encoded in the time dependence of the fluorescence signal, the setup only requires a single detector thereby eliminating the need for complex off-chip optics. The fluidics of the chip is designed carefully to prevent fluid sample contamination among the channels. The inlets for the analyte are located on one side of the device, and combine into a single outlet only after crossing the MMI waveguide. Fluidic reservoirs are attached on the inlets and outlets to create an interface for the fluidic delivery, and negative pressure is applied to the outlet reservoir to achieve unidirectional flow. The full chip with attached reservoirs is shown in Fig.1b, inset.

The MMI waveguide supports numerous waveguide modes with different propagation constants and allows them to interfere with each other as they propagate along the structure^22^. At certain propagation distances where the relative phases of these modes match up constructively, well-defined spot patterns are created. Here, we design the MMI and the intersection locations of the 3 liquid channels such that well-defined, but distinct, integer numbers of spots are generated at a certain wavelength in each channel. The dependence of the spot number (*N)* on distance from the beginning of the MMI (*L*) and the excitation wavelength λ, can be derived from standard MMI theory^22^ and is given by:

1 $$N(\lambda ,L)=\frac{{n}_{C}{w}^{2}}{\lambda L}$$

where *w* is the effective MMI-WG width (here: 75 µm) and *n*_*c*_ the effective refractive index of the MMI-WG (here: 1.46). When λ = 633 nm; 8, 6, and 4 well-defined spots are produced at the MMI lengths of *L*_1_ = 1676µm, *L*_2_ = 2243 µm, and *L*_3_ = 3350 µm respectively. This provides a way for *spatial* multiplexing. In addition, we designed the chip such that we can choose another wavelength that generates a second set of distinct, integer spot numbers in each channel, adding the option for *spectral* multiplexing. For the exact same device, using Eqn. (1), we find that by choosing λ = 520 nm as the excitation wavelength, we can create 10, 7, and 5 well-defined spots at the same MMI lengths. Figure 1c confirms this by showing the spot patterns in the three channels when filled with a mixture of quantum dots which are fluorescently excited at the two design wavelengths. In this way, from a single MMI, both spatial and spectral properties of MMI can be combined to create 6 distinct spot patterns for multiplexing.

Another important part of the optical design is the efficient collection of signals from all the channels. We have carefully designed a Y-coupler with optimized dimensions in order to achieve high and equal signal collection efficiency from all channels. Beam propagation software (FimmWAVE, Photon Design) was used to minimize propagation loss in the curved waveguide sections. Based on these simulations, the fan-out angle of the outermost collection arms is kept small (~8°) while keeping the waveguide short. Moreover, it is important to balance the signal strength from the first and third channels to increase our detection sensitivity. The MMI waveguide has a finite propagation loss which results in a smaller excitation power in the third channel, as compared to first and second channels. However, the emitted signal intensity from a fluorescent particle depends on the excitation power of each individual spot, which is given by the total excitation power at that channel, divided by *N*. Since the total power is divided amongst a smaller number of spots in the third channel as compared to the first channel, the signal intensity per peak is higher. This property, therefore, compensates for the propagation loss in the MMI waveguide, leading to comparable signal intensities from all three channels. For this reason, in order to achieve similar collection efficiencies, the angles of the Y-coupler are kept the same for the first and the third collection arms

## Results

### Characterization of Spot Patterns

In order to characterize the excitation spot patterns, 3 intersecting fluidic channels were filled with solutions containing a mixture of quantum dots which have a broad excitation spectrum. The MMI section was excited with the tunable laser source, and the wavelength is tuned to optimize the fidelity of the spot patterns. The most well-defined patterns were found at λ = 520 nm and λ = 633 nm. Their fluorescence when imaged with an overhead CCD camera shows the desired well defined excitation spots for all three channels and 2 separate wavelengths (Fig. 1c).

### Spatial Multiplexing

For the first demonstration of a spatially multiplexed virus detection assay, red (Dylight 633) fluorescently labeled deactivated H2N2 virus solution was pipetted into the three input reservoirs. The solutions were then pulled through the 3 different liquid-core analyte channels and past the MMI excitation areas via negative pressure applied at the output reservoir. The laser source was tuned to 633 nm to excite the labeled viruses. As the targets traveled down the liquid core waveguides, they passed through 4, 6, or 8 different excitation spots, depending on the channel they were in. The resulting fluorescence signals were captured by the liquid-core waveguides, collected by the solid-core collection waveguides and combined into a single output waveguide by using the 3 × 1 Y-coupler (see Fig. 1a). The excitation wavelength was eliminated by a notch filter, before the time dependent fluorescent signal was finally detected by a single-photon-counting detector.

**Figure 2:**
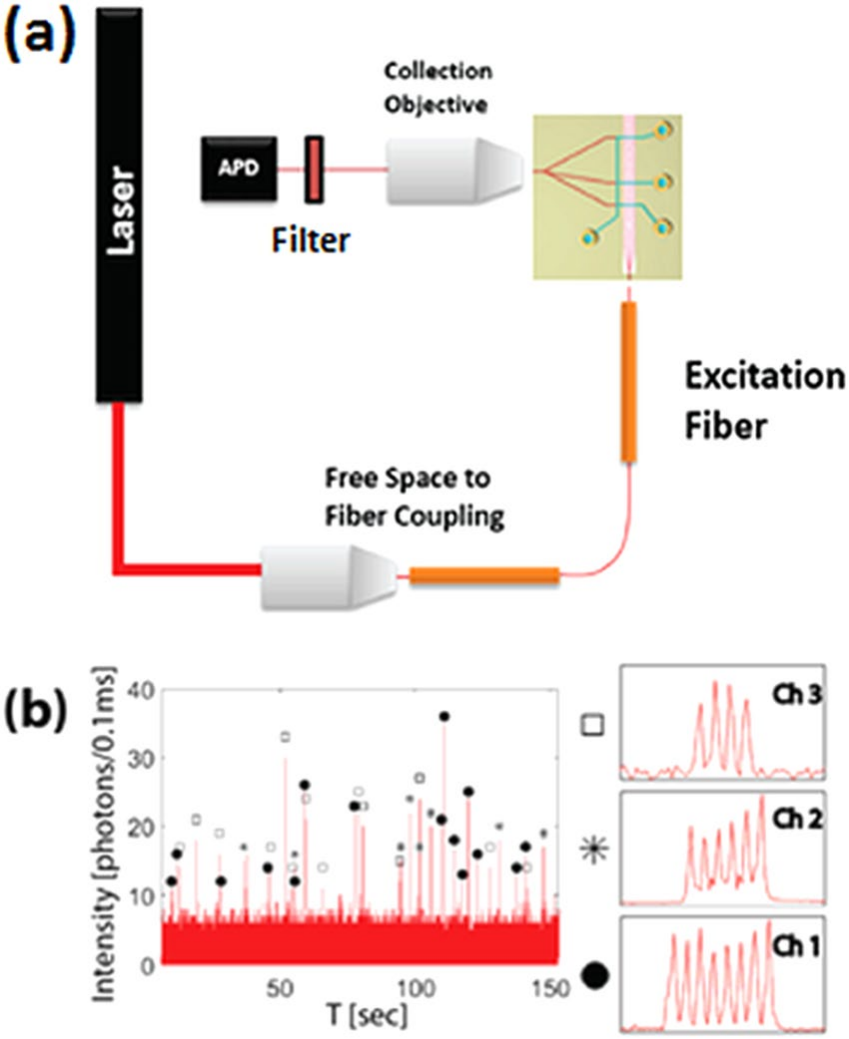
(**a**) Experiment setup showing optical excitation and collection paths. (**b**) Fluorescence signal collected from single H2N2 viruses excited with λ = 633 nm only. The zoomed-in images show the single virus detection for different numbers of spots (4, 6, and 8) originating from 3 separate channels.

Figure 2b shows the fluorescence trace where each particle signal, defined as signal values above a threshold of photon intensity of 10 photons/0.1 ms, is the signal emitted from a single labeled H2N2 virus. Each signal, thus identified, is then zoomed in to reveal a unique peak pattern. Within each pattern, distinct peaks are clearly visible, illustrating both the high quality of the MMI pattern and the sensitivity of the optofluidic chip to single virus particles. By simply counting the number of peaks of each particle signal, we were able to uniquely identify the channel from which the virus originated. More advanced signal processing methods can also be applied to enhance the signal-to-noise ratio and hence increase the fidelity of identification^23^. It should be noted that the fluctuation in the peak height can arise due to various sources – variation due to changes in a particle’s cross sectional position as it flows across the MMI channel, or optical variations introduced due to imperfect fabrication in the MMI section, or imperfect fiber alignment. However, particle identification only depends on the number of peaks in the pattern, or the temporal spacing ∆T between the peaks, which are very robust against the factors mentioned above.

This multi-channel multiplexing ability can be applied in different ways. For example, a test can be run for samples from three different patients to determine which ones contain the target virus and therefore used to screen for a disease in the three different patients simultaneously. Alternatively, different types of viruses can be placed in the three different reservoirs in order to do simultaneous detection of different targets with a single color. Finally, a single sample can be split and analyzed three times faster. Clearly, the multiplexing ability of the triple-core chip allows for versatile sample analysis and can therefore be adapted for different clinical needs.

### Spectral-Spatial Multiplexing

Next, we demonstrated the capability for joint (spectral and spatial) multiplexing, which significantly increases the multiplexing capacity of the device in a highly scalable manner. For the demonstration of a joint multiplexed virus detection assay, H2N2 deactivated virus type was labeled with red (Dylight 633) fluorescent dye and H1N1 deactivated virus type was labeled with green (Dylight 550) fluorescent dye. They were mixed and pipetted into the three input reservoirs and the flow was initiated via negative pressure applied at the output reservoir as before.

**Figure 3:**
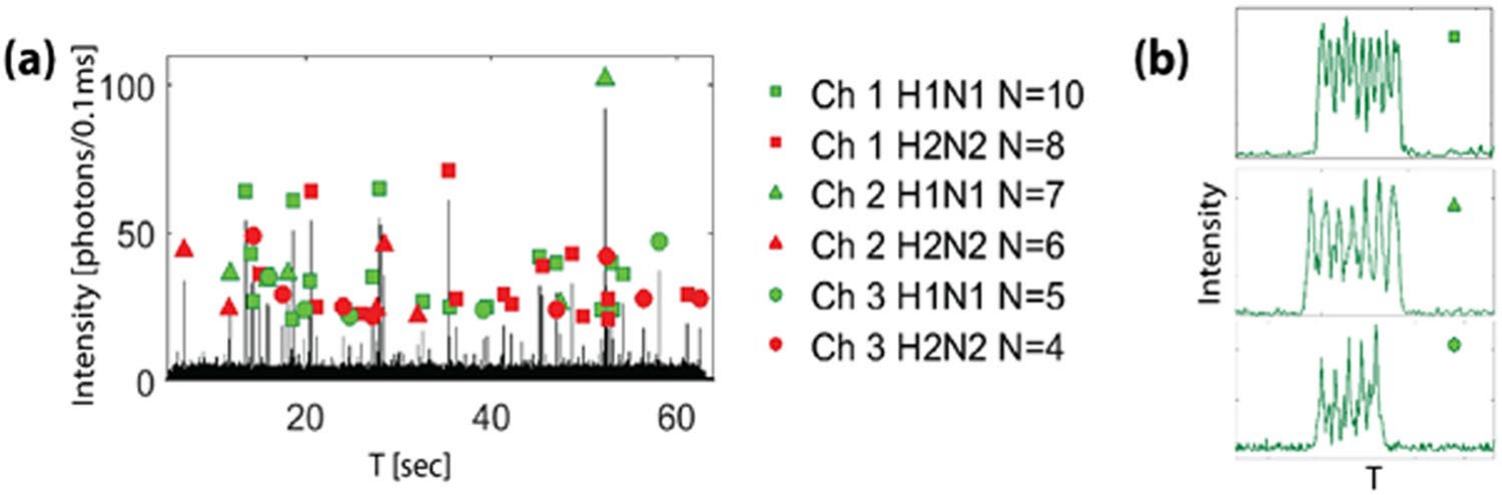
(**a**) Fluorescence signal collected from viruses in three channels, excited with both wavelengths λ = 520 nm and λ = 633 nm. They are labeled based on the number of peaks they have. (**b**) Zoomed in fluorescence signal of single virus detection for H1N1, showing three distinct numbers of peaks (10, 7 and 5), and coming from 3 separate channels.

Figure 3a shows a fluorescence signal trace in which a mixture of both viruses was excited simultaneously at both 520 and 633 nm. Each peak corresponds to a single H1N1 or H2N2 virus from either of the three different channels, and collected together from a single collection waveguide. By counting the number of peaks in each signal above the background threshold of 10 photon counts/0.1 sec, we were able to distinguish between the 6 separate multiplexing routes, and identify both the channel and the type of virus. Figure 3b shows the individual virus signals generated from only H1N1 viruses in the 3 separate channels. These show the correct number of peaks, distinct and complementary to the red-labeled H2N2 viruses (Fig. 2).

Automated spot counting with a custom-written program identified with 97% accuracy as compared to manual inspection during identification, mostly due to distorted signals caused by flow variations. Furthermore, the velocities and the concentrations of the sample can be extracted from the dimensions of the excitation volume and the time difference between the peaks, respectively. The average velocity is found to be 0.87 cm/sec and the concentration of all viruses is estimated to be 6 × 10^5^ particles/mL, which is in good agreement with previous assays at clinically relevant concentrations^21^. Combining multiple wavelengths with multiple channels enables detection and differentiation of many different target samples based on the number of spots, in the same experiment, without the need for a spectral filter to separate signals.

### Spectral-Spatial Multiplexing with Six Different Influenza Strains

Finally, we demonstrate simultaneous screening for six different strains from a standard influenza panel^6^ in parallel by using the spatial-spectral capability described above. For these experiments, deactivated virus solutions of H1N1, H2N2 and H3N2 virus strains were labeled with green (Cy3) fluorescent dye, while those of Adenovirus (AV), and Parainfluenza PIV1 and PIV3 viruses were labeled with red (Cy5) fluorescent dye. Three separate virus mixtures were then prepared from the above fluorescently labeled solutions as follows: Solution A with virus strains H1N1 and PIV3; Solution B with virus strains H2N2 and PIV1, and Solution C with virus strains H3N2 and AV. Therefore, each solution A, B and C contained one virus strain labeled with the red fluorescent dye, and one virus strain with green fluorescent dye. Solutions A, B and C were then pipetted into input reservoirs for liquid channels 1, 2 and 3 respectively, with a negative pressure applied at the output reservoir to drive flow in the three liquid channels. The MMI was then excited by laser light at wavelengths of λ = 525 nm and λ = 633 nm to generate the desired six spot patterns in the three intersecting liquid channels.

**Figure 4:**
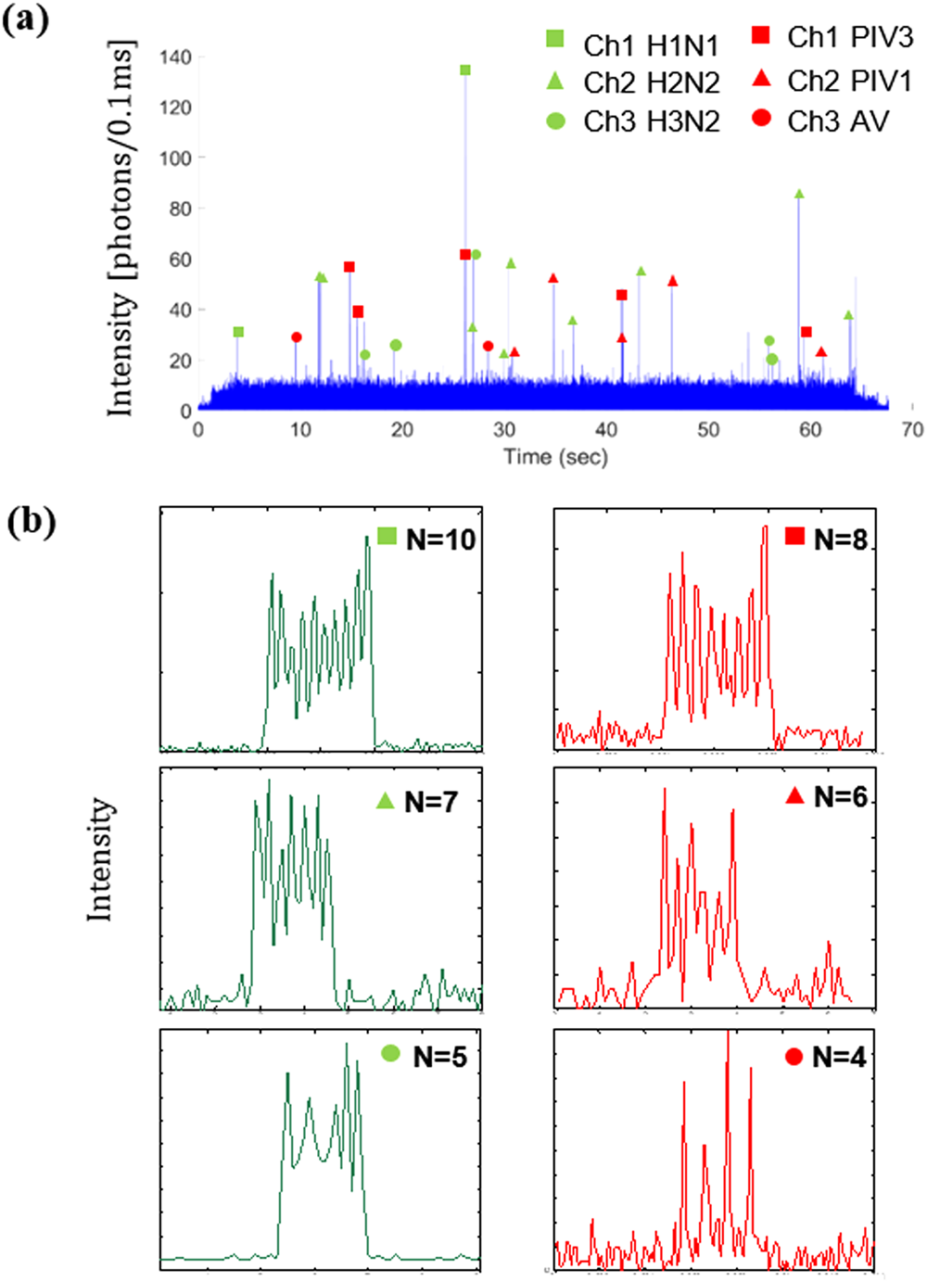
(**a**) Fluorescence signal trace collected from viruses in the three intersecting channels with each channel containing one virus strain labeled with a green (Cy3) dye, and one virus strain labeled with red (Cy5) dye, and simultaneously excited with wavelengths λ = 525 nm and λ = 633 nm. They are subsequently identified uniquely by determining the number of spots in each individual peak. (**b**) Peak patterns observed for each type of virus strain with the green excitation (λ = 525 nm) generating N = 10, 7 and 5 spots and red excitation (λ = 633 nm) generating N = 8, 6 and 4 spots at channels 1, 2 and 3 respectively.

Figure 4a shows the resulting fluorescence signal trace from the mixture of all six viruses. Each individual signal above a threshold of 12 photon counts/0.1 sec was considered a positive detection event, corresponding to a single virus. The distinct peak pattern served as an identifier for that particular virus type. Peak patterns with N = 10, N = 7 and N = 5 confirmed the presence of H1N1, H2N2 and H3N2 virus strains (labeled with Cy3) respectively, while peak patterns with N = 8, N = 6 and N = 4 confirmed the presence of PIV3, PIV1 and AV virus strains (labeled with Cy5) respectively. It should be noted again that all the signals were collected from a single collection channel without any spectral separation into each color channel. Figure 4b shows the individual peak patterns that were generated for each virus type. The maximum SNR for these peak patterns was found to be 20, with an average SNR of 10.5, which is comparable to the maximum SNR of 38 reported for detecting influenza viruses using shifts in optical microcavity resonances^24^. For this assay, the peak number was determined using two independent methods, described in more detail in Supplementary Information (SI). Only if both methods produced the same result for the peak number, was the virus considered as identified. While this results in a somewhat lower identification rate than the single-step identification used in Fig. 3, it represents a more robust and high fidelity identification of each virus type, in line with the requirements for real-world applications. The fidelity of this multiplex assay can be further improved in the future by optimizing the optical performance of the device and the analysis procedure.

## Discussion

In summary, scalable joint (spatial and spectral) multiplexed detection of influenza viruses was achieved on an optofluidic chip consisting of triple liquid-core waveguides intersecting a single solid-core MMI waveguide. We successfully detected and identified single viruses from 6 different strains simultaneously and distinguished among them based on the number of fluorescence peaks they generated. This approach enables scaling up MMI-based multiplexing to large target numbers. It facilitates simultaneous analysis of multiple bioparticles of different types on a single, fully integrated platform while taking advantage of established fluorescence technologies. It should be noted that at high concentrations where multiple particles are excited too close together to be temporally separated, it might not be always be possible to distinguish each particle individually. However this is not a concern at clinically relevant concentrations which are low enough to allow for single bioparticles to be probed individually by the excitation volume at a given time. Further, if the concentration of the sample was higher than the single molecular regime for our device, the sample can be diluted accordingly to suitable concentration ranges.

By incorporating the multi-color labeling approach detailed in^21^ we can further increase the capacity of multiplexing while using minimal number of different fluorescent dyes and color channels. For the present example, adding co-labeling with two dyes translates to nine possible targets for only two excitation colors. The optofluidic platform demonstrated here can also be integrated with a dedicated microfluidic layer for sample processing and distribution as shown in previous work^19,20,25^. This platform therefore serves as a powerful diagnostic tool by allowing the single-molecule detection and identification for variety of pathogens and biomarkers.

## Materials and Methods

### Experimental Setup

The experimental setup implemented for multiplexed virus detection can be seen in Fig. 2a. A white light laser (NKT SuperK Extreme) passing through a multi-line tunable filter with a narrow bandwidth (NKT SuperK Select) is coupled into a single-mode fiber. This allows us to precisely tune the source to a desired wavelength across a wide spectral range, and introduce multiple laser lines at the same time into the fiber. A single-mode fiber is butt-coupled into the single-mode excitation waveguide, which feeds into the center of the MMI section.

Fluorescently labeled viruses passing through the MMI excitation volume are excited by the spatially distributed MMI spot pattern and thus generate multiple fluorescent peaks per virus in the collected time domain signal. These signals are captured by the liquid-core waveguide orthogonal to the excitation, transmitted into the collection solid-core waveguides, which then combine into a single solid-core waveguide via the Y-coupler. An avalanche photodiode (Excelitas) is used to detect the fluorescence signal after removing the excitation wavelengths with a set of filters (Edmund Optics 633 nm Notch Filter and Semrock 532 nm Long Pass). No spectral filters are required to separate the emission colors.

### Fluorescent Labeling of Influenza Viruses

For the first set of experiments to demonstrate spatial multiplexing and joint multiplexing with two strains of viruses, purified, UV-inactivated A/2/Japan/305/57 H2N2 Human Influenza Virus (Advanced Biotechnology Inc.) was labeled with Dylight 633 and β-Propiolactone-inactivated A/PR/8/34 H1N1 Human Influenza Virus (Advanced Biotechnology Inc.) was labeled with Dylight 550 NHS ester-activated dyes according to manufacturer specifications (Thermo Scientific). Unbound dye was removed by column chromatography using a PD MiniTrap G-25 column and 1 x PBS elution buffer (GE Healthcare Life Sciences), and efficient labeling was verified by TIRF microscopy.

For our final set of experiments with six-fold multiplexing, six inactivated virus samples from the influenza panel^6^, were purchased for testing - β-Propiolactone-inactivated A/PR/8/34 (H1N1), UV-inactivated A/2/Japan/305/57 (H2N2) (Advanced Biotechnologies Inc.), UV-inactivated purified Parainfluenza Viruses 1 and 3 and Adenovirus (ViraSource), as well as Formalin-inactivated A/Aichi/68 (H3N2) (Charles River). Viruses were labeled using NHS-activated Sulfo-Cyanine fluorophores (Cy3 and Cy5) according to manufacturer instructions (Lumiprobe). Briefly, virus samples were added to aliquots of dried Cyanine dyes and allowed to incubate at room temperature for 2 hours. Samples were then separated from free dye using PD MiniTrap G-25 gel filtration columns (GE Healthcare). Fractions were collected dropwise and tested for fluorescent virus and free-dye background on a Leica SP5 confocal microscope to determine if there was efficient labeling of the virus particles. Once identified, the viral fractions were aliquoted and flash-frozen via liquid nitrogen for later use.

### Device Fabrication

The devices were created on top of a < 100 > oriented Si substrate. The mask alignments were designed to cleave across the (100) plane in order to produce clean unpolished facets. Six alternating dielectric layers of SiO_2_ (n = 1.47) and Ta_2_O_5_ (n = 2.107) were then sputtered over the whole wafer to thicknesses of 265 nm and 102 nm respectively, forming the ARROW layer stack, which acts as the substrate in subsequent fabrication steps. The three 6 × 12 µm hollow-core microchannels, which carry the biological sample in the final device, were defined using standard lithography procedures for SU-8^26^, and then hard baked at a maximum temperature of 250 °C to withstand further processing. A self-aligned pedestal was defined as described in^27^ to improve the structural integrity of the hollow cores. This step protects the SU-8 core while etching the ARROW dielectric stack. Once the pedestal was defined, a 6 µm thick PECVD oxide layer was deposited on top using a low stress recipe to reduce potential core cracking^28^. The MMI and collection waveguides were then patterned by photolithography and etched with an ICP/RIE etcher to create 3 µm tall rib waveguides. The SU-8 cores were exposed at the corners by removing the oxide with buffered hydrofluoric acid and then placed in a strong acid to remove the SU-8, hollowing out the liquid-core channel^29^.

### Data availability statement

Requests for materials and data should be addressed to the corresponding author (Prof. Holger Schmidt)

## Electronic supplementary material

Supplementary information for Scalable Spatial-Spectral Multiplexing of Single-Virus Detection Using Multimode Interference Waveguides
