# Supplementary material for: Scalable Spatial-Spectral Multiplexing of Single-Virus Detection Using Multimode Interference Waveguides
Source: Sci Rep. 2017 Sep 22;7:12199. doi: 10.1038/s41598-017-12487-0 (PMC5610187; doi:10.1038/s41598-017-12487-0)
Supplement: Supplementary file 1 — Supplementary information for Scalable Spatial-Spectral Multiplexing of Single-Virus Detection Using Multimode Interference Waveguides [file 41598_2017_12487_MOESM1_ESM.pdf]

Supplementary Information

Title: Scalable Spatial-Spectral Multiplexing of Single-Virus Detection Using Multimode Interference Waveguides

Author List and Affiliations: Damla Ozcelik[1], Aadhar Jain[1], Alexandra Stambaugh[1], Matthew A. Stott[2], Joshua Parks[1], Aaron Hawkins[2], Holger Schmidt[1*]

[1] School of Engineering, University of California Santa Cruz, 1156 High Street, Santa Cruz, CA 95064 USA.
[2] ECEn Department, 459 Clyde Building, Brigham Young University, Provo, UT 84602 USA.
[*]corresponding author. Email: hschmidt@soe.ucsc.edu, phone: 831-459-1482

Here we discuss in detail the method of analysis we use to determine the number of excitation spots in MMI waveguide generated spot patterns and thus uniquely identify the virus strain being detected. We used a combination of two different methods to robustly and accurately ascertain the number of peaks in each signal, and thus uniquely identify the type of virus being detected. After identifying a multi-peak signal ("signal") through thresholding in the fluorescence time trace *F(t)* from the photodetector, we first determine the characteristic time spacing between peaks **ΔT** for the peak pattern via the autocorrelation of *F(t)* as detailed in [1]. This provides us with an accurate estimate of the time spacing between each peak in the pattern and is unique to each pattern (Fig. S1 (a)).

In the first identification method, the number of spots are directly determined by simply dividing the total width of the signal T_{tot} (Fig. S1 (b)) by the characteristic time Δ T. Since Δ T is the time spacing between peaks, it should evenly divide T_{tot} and thus provide us with the number of peaks in the pattern:

$$N_{spots,1} = T_{tot}/\Delta T \qquad \text{(S1)}$$

For the 2nd method, we applied a modified version of the 'shift and multiply' algorithm detailed in [2], which leads to an enhancement of the signal to noise ratio of a multi-spot pattern. Unlike in [2] however, we assume no *a priori* knowledge of the number of peaks in the pattern and we compute the following normalized product $S_j(t)$:

$$S(t, \Delta T) = \prod_{i=0}^{j} F(t - i.\Delta T) / (\mu + \sigma)^{j} \tag{S2}$$

where F(t) is the time-dependent fluorescence signal, μ and σ are the mean and standard deviation of the noise, and j indicates the number of iterations of 'shift and multiply'. The normalization factor $(\mu + \sigma)^j$ in the denominator ensures that S_j (t) decreases whenever j becomes greater than the number of peaks.

$S_j(t)$ is calculated for all possible values of j (here3 to 9) that correspond to all possible spot numbers (here 4 to 10) , and the value of 'j' – j_{max} - at which $S_j(t)$ becomes maximum is determined. The number of peaks in the pattern is then given by:

$$N_{peaks,2} = j_{max} + 1 \tag{S3}$$

Fig S1(c) shows a typical plot of the product $S_j(t)$ versus number of iterations j, for the signal with 5 peaks shown in Fig S1(b) . As we increase j, the value of $S_j(t)$ also increases as the peaks in the fluorescence signal are sequentially multiplied by each other. However once j becomes greater or equal to the number of spots in the excitation pattern (in this case when j>4), $S_j(t)$ starts to decrease with increasing j since for all subsequent iterations, it is only multiplied by the noise while simultaneously being normalized by maximum noise intensity**.**

If both independently computed $N_{spots,1}$ and $N_{spots,2}$ are found to be equal, then we assign the number of peaks in the pattern as $N_{peaks} = N_{peaks,1} = N_{peaks,2}$ and use it to uniquely identify the virus in question. If, on the other hand, we find a discrepancy between $N_{peaks,1}$ and $N_{peaks,2}$ that signal is said to be undetermined**.**

REFERENCES:

1. Ozcelik, D. *et al.* Optofluidic wavelength division multiplexing for single-virus detection. *Proc. Natl. Acad. Sci. U. S. A.* **112,** 12933–7 (2015).
2. Ozcelik, D. *et al.* Signal-to-noise enhancement in optical detection of single viruses with multi-spot excitation. *IEEE J. Sel. Top. Quantum Electron.* **PP,** 1–1 (2015).

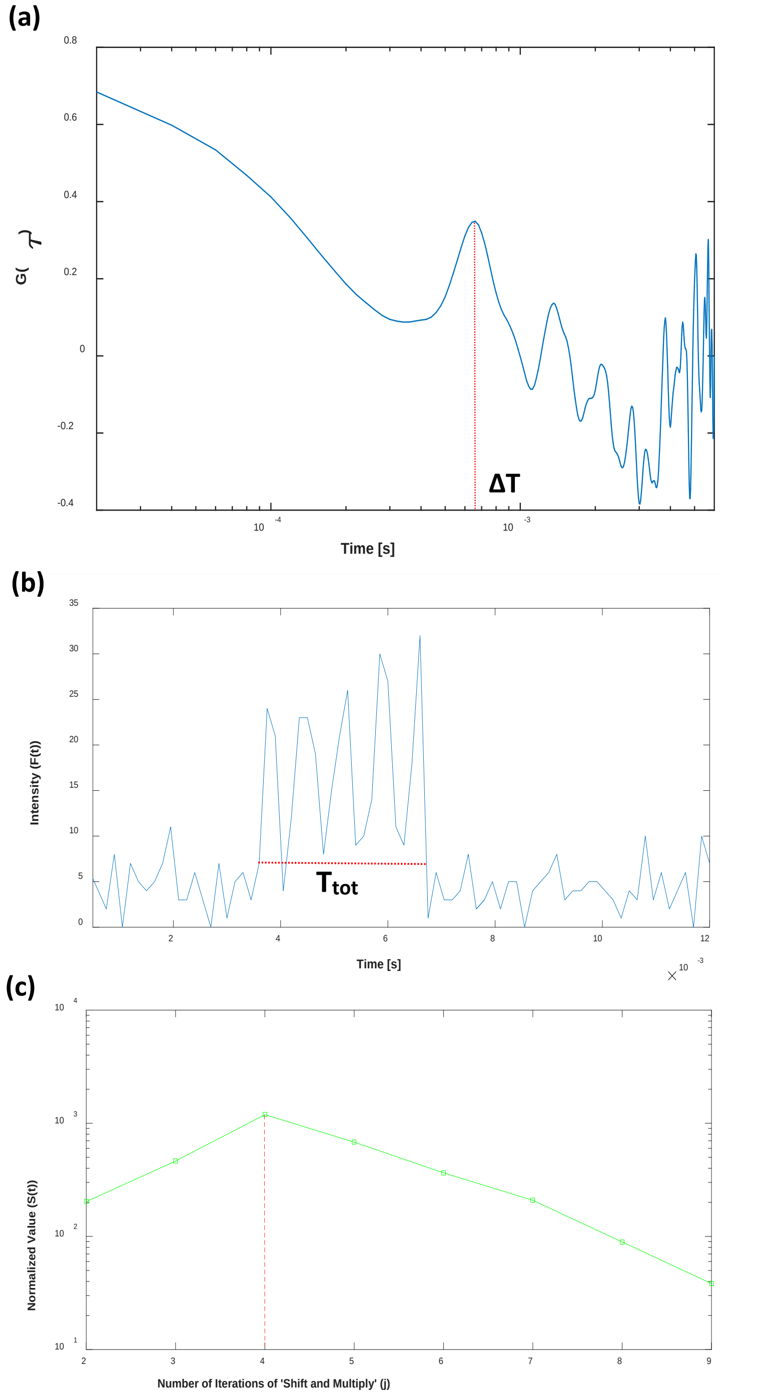

Figure S1 (a) Autocorrelation function of the signal F(t). The first peak as indicated determines the characteristic time period ΔT of the signal. (b) Signal intensity F(t) with respect to time of a typical spot pattern. The peak width T_{tot} is shown. (c) Plot of normalized value $S_j(t)$ vs number of iterations 'j'. In the example shown, since number of spots is 5, the graph maximizes at j=4, and then decreases with further increase in j.
